# Are node-based and stem-based clades equivalent? Insights from graph theory

**DOI:** 10.1371/currents.RRN1196

**Published:** 2010-11-22

**Authors:** Jeremy Martin, David Blackburn, E. O. Wiley

**Affiliations:** ^*^University of Kansas; ^†^University of Kansas Biodiversity Institute and ^‡^The University of Kansas

## Abstract

Despite the prominence of “tree-thinking” among contemporary systematists and evolutionary biologists, the biological meaning of different mathematical representations of phylogenies may still be muddled. We compare two basic kinds of discrete mathematical models used to portray phylogenetic relationships among species and higher taxa: stem-based trees and node-based trees. Each model is a tree in the sense that is commonly used in mathematics; the difference between them lies in the biological interpretation of their vertices and edges. Stem-based and node-based trees carry exactly the same information and the biological interpretation of each is similar. Translation between these two kinds of trees can be accomplished by a simple algorithm, which we provide. With the mathematical representation of stem-based and node-based trees clarified, we argue for a distinction between types of trees and types of names. Node-based and stem-based trees contain exactly the same information for naming clades. However, evolutionary concepts, such as monophyly, are represented as different mathematical substructures in the two models. For a given stem-based tree, one should employ stem-based names, whereas for a given node-based tree, one should use node-based names, but applying a node-based name to a stem-based tree is not logical because node-based names cannot exist on a stem-based tree and visa versa. Authors might use node-based and stem-based concepts of monophyly for the same representation of a phylogeny, yet, if so, they must recognize that such a representation differs from the graphical models used for computing in phylogenetic systematics.

**Introduction**

“Tree-thinking”, using phylogenies to understand evolutionary relationships, name clades, and understand evolutionary transformations and biogeography, is now ubiquitous in systematics and evolutionary biology and is making its way quickly into the educational and public realms (e.g., [1]; [2]; [3]). But the biological interpretation of the precise mathematical notion of a tree often remains unclear ([4]). We argue that the two dominant representations of phylogenies used today (node-based and stem-based) are mathematically equivalent, but not identical. We then argue that if these two forms of trees are not considered separate and distinct representations of the same information, then biological interpretations of trees and evolutionary transformations may become confused.

   In the Willi Hennig Memorial Symposium, held in 1977 and published in Systematic Zoology in 1979, David Hull expressed the concern that “uncertainty over what it is that cladograms are supposed to depict and how they are supposed to depict it has been one of the chief sources of confusion in the controversy over cladism” ([5], p.420). Early disagreements concerning the differences between cladograms and phylogenetic trees were largely generated by such differences ([6]; [7]; [8]; [9] [10] [11]). This debate has largely subsided, yet the importance of representing phylogenies and interpreting their biological meaning remains. The purpose of this article is to compare what we believe to be the two most commonly used tree models of phylogenetic relationships, namely node-based and stem-based (or branch-based) trees, using the mathematical techniques of graph theory.  We consider node-based and stem-based trees to be representations of phylogenies as both explicitly model hypotheses of common  ancestry. We assert that it is imperative to understand the mathematical relationships between these two graphical representations of phylogenies to make meaningful biological statements. In doing so, we aim to finally lay to rest the “uncertainty” observed by Hull thirty years ago.

   The vertices of a node-based tree represent taxa (sampled or inferred), while its edges model ancestry relationships. For example, if the tree represents the results of a phylogenetic analysis, then the tips of the tree are nodes and internal nodes represent inferred common ancestors. By contrast, in a stem-based tree, both sampled and inferred ancestral taxa are modeled by edges, while vertices correspond to speciation events.  These two models are isomorphic (as that term is used in mathematics) but not equal: that is, they carry exactly the same information about ancestry, but it is encoded in two different ways.  To make this explicit, we give a simple algorithm that constructs a unique node-based tree for every stem-based tree and vice versa. While some might see as frivolous the demonstration that these two tree models are equivalent, the relationship between these two representations has important repercussions for evaluating the biological meaning of trees. Thus, we provide an explicit example of the need for distinction between these representations through a discussion of how the phylogenetic concept of monophyly is represented in each graphical model.  

**Some basic graph theory**

   Mathematically speaking, all of the diagrams we shall consider are *graphs*: they are finite structures built out of *vertices* (sometimes called * nodes*) and *edges*, in which each edge connects two vertices (see [12]) for background.  A graph is usually represented by drawing the vertices as dots and the edges as line segments.  Frequently, the vertices and/or edges are labeled with names, numbers, or other data.  Graphs provide a simple and powerful tool to model and study phylogenetic and synapomorphic relationships between taxa (and many other structures). Utilizing graphs as representations of this sort has a long history in the study of organismal evolution with famous early examples including the sole figure in Charles Darwin's ([13]) *Origin of Species*. However, one must be very careful to keep track of what the individual vertices and edges are supposed to mean, particularly when there is more than one way to represent the same biological data in a graph. Until the techniques promoted by Hennig ([14]) gained wide use, graphs were essentially cartoons sketched out by hand rather than representing the output of an analytical inference in the sense that phylogenies are now typically used. With the advent of phylogenetic analyses, the representation used for trees of evolutionary relationships became non-trivial. Before proceeding, we mention a few basic facts and terms from graph theory, so as to have a unified mathematical language with which to work. We will introduce more technical material later, as needed.

   We will primarily be concerned with graphs that are *trees*.  Mathematically, a tree is a graph *T* containing no closed loops; intuitively, if you walk along the edges from vertex to vertex, the only way to return to your starting point is to retrace your steps. Put in an evolutionary context, this means that trees in this sense cannot have reticulations within them. If we designate one vertex *r* as the *root* of *T*, then every edge connects a vertex *x* that is closer to *r* with a vertex *y* that is further away.  In this case, we say that *x* is the *parent* of *y*, and it is often convenient to regard the edge between them as a directed edge (or *arc*) pointing from *x* to *y*, represented by the symbol *x*→*y*.  Every vertex in a tree has a unique parent, except for the root, which has no parent.  An immediate consequence is the useful fact that every tree with *n* edges has * n*+1 vertices, and vice versa, though, of course, several different vertices may share a common parent (i.e., a polytomy).

   The *ancestors* of a vertex are its parent, its parent’s parent, its parent’s parent’s parent, and so on.  Equivalently, we might say that an edge * x→y* is an ancestor of another edge *a*→*b* if *y* is equal to, or an ancestor of, *a*.  A *lineage* (or * ancestral lineage*)of a vertex *x* is the complete list of vertices that are ancestors of *x* and are descendants of, or equal to, some other vertex *y*.  If *y * = root(*T*), then this list is called the *total lineage* of *x*.  It is important to note that in a tree with a root the choice of a root vertex, together with the topology of the tree, completely determines all ancestry relationships.

   A *subtree* of a tree *T* is a tree *U* all of whose vertices and edges are vertices and edges of *T* as well.  This is equivalent to saying that *U* can be formed by removing some vertices and edges from *T*.  If in addition *T* is a rooted tree, then *U* inherits its “ancestor-of” relation from *T* as well.  A *proper subtree* of a rooted tree is a subtree that consists of a vertex and all its descendants.  A proper subtree is uniquely determined by its root vertex, so there are exactly as many proper subtrees of *T* as there are vertices.

   Trees are well suited for modeling phylogenetic relationships between species or taxa, in which each species or taxon has a unique parent.  Uniqueness is vital; a tree in the sense that we use it here cannot model reticulations, such as tokogenetic relationships in a sexually reproducing species or hybridization events between two different species.   

**Stem-based trees**

**Figure:**
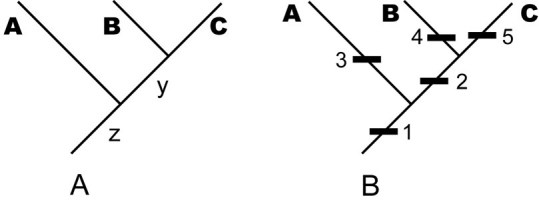
**Fig. 1.** A. An example of a stem-based tree, indicating the evolutionary relationship among the sampled taxa A, B, C and their unsampled, but inferred, ancestral species y and z. — (B) The same tree with character data shown (the names of the internal edges have been omitted for clarity).  In each case, taxon names are displaced from the leaf position to emphasize that the edge is the taxon.

By the term * stem-based tree, *we mean a tree that models (hypothesized) phylogenetic relationships among taxa by depicting taxa as edges and speciation events as vertices.  For instance, in the tree in Fig. 1A, the terminal edges, labeled A, B, and C, represent named taxa; that is, larger groups of individual organisms represented by sampled specimens.  The internal edges, labeled *y* and *z*, represent ancestral lineages needed to account for the terminal taxa under the paradigm of descent with modification.  The vertices represent speciation events, in which the edge below the vertex is the common ancestor and the edges above it are descendants.  Mathematically, the edge *y* is the youngest common ancestor of edges B and C*. * Biologically, moving up the tree represents moving forward in time, so the edge *y* represents a lineage of common ancestors of the sampled taxa Band C, occurring before the speciation event that distinguishes B and C and after any previous speciation events.*  * Thus the total lineage of a species (or, more properly, a hypothesis of its lineage) is represented by a chain of edges starting with the species itself and moving down the tree towards the root vertex, which necessarily has only one edge emanating from it—representing the common ancestor of all sampled taxa. 

   We frequently refer to the internal edges as “hypothetical” ancestors. However, under the paradigm of evolution, there is nothing more hypothetical about these edges than there are about the named taxa represented by specimens. If the inferred tree is correct, then these ancestral taxa represented by these edges must have existed. Under the evolutionary paradigm, the extent to which we treat named taxa (A, B, C) as real entities of descent with modification is the extent to which we treat internal lines as symbolizing real ancestors. They are not “hypothetical”; they are simply unsampled and inferred (or, conceivable especially in systematics of fossil organisms, unrecognized or misidentified as descendant species).

   In Fig. 1B, we have added more information to the tree.  Each numbered black rectangle represents an evolutionary character hypothesized to be fixed (sensu [15]) somewhere in the lineage represented by the edge to which the rectangle is attached.  (The placement of the rectangle within an edge does not matter; for example, the tree in Fig. 1B does *not *assert that apomorphies 3, 4, and 5 became fixed at different times just because they are shown at different heights on the page.  Moreover, one cannot draw inferences about when characters originated; for example, it is possible that character 2 originated in lineage z before character 1, but went extinct in other lineages (such as A) and became fixed only in the common ancestor y of B and C.)  

**Node-based trees**

   Hennig ([14]) used the symbology of Gregg ([16]), which Gregg apparently derived from Woodger ([17]).  In a node-based tree, taxa are represented by vertices, not by edges.  An edge of a node-based tree does not represent a lineage or anything else occurring in nature. Rather, an edge simply represents a relationship among two vertices, or, in phylogenetic parlance, the hypothesis of a relationship.  Specifically, an edge between a parent vertex X and a child vertex Y represents the hypothesis that  X is an ancestor of Y. This node-based tree representation is fairly intuitive (at least to us) and likely how most practicing evolutionary biologists interpret phylogenies.

**Figure:**
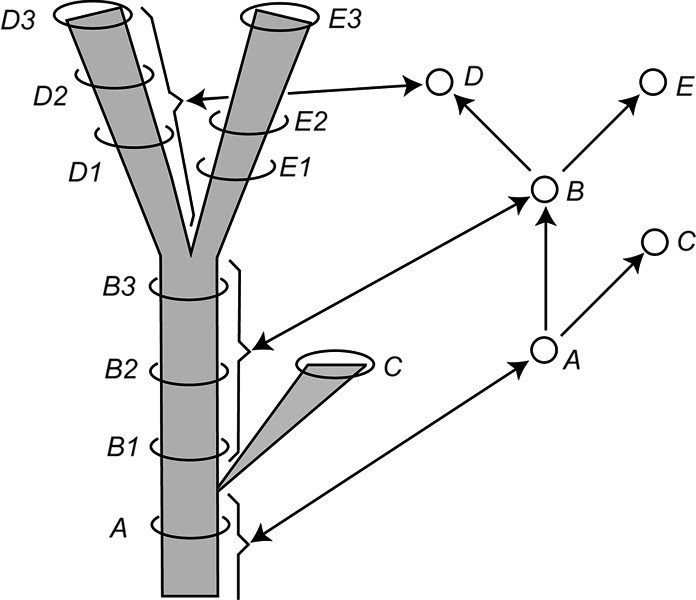
**Fig. 2.** Modified version of Figure 14 of Hennig (1966, p. 59) entitled “The species category in the time dimension.” Left: a stem-based tree. Letters are symbols for species and the number applied to the letters are labels for samples of each species considered at a particular time period. Right: a node-based tree with single-headed arrows symbolizing relationship statements and circles representing species. Note the correspondence between the lineages on the left and the circles on the right, as shown by the brackets and double-headed arrows for selected lineages and vertices. 

  Fig. 2 is redrawn from Hennig ([14]) and portrays the relationships among samples of an evolving clade in two ways. The left-hand side of Fig. 2 portrays a stem-based tree with lineages represented by edges (species to Hennig) and sampled populations of these lineages placed in time with circles (B1, B2, etc.).  Vertices represent speciation events. The right-hand side of Fig. 2 shows the node-based tree corresponding to the stem-based tree on the left-hand side.  Here the taxa are represented by vertices (population samples being completely ignored).  The edges represent phylogenetic, not phenetic, relationships between these species (i.e., genealogical relationships based on synapomorphies rather than similarity relationships based on a metric or idea of overall similarity). Hennig ([14]) makes this clear in a number of diagrams (see his Figs. 4, 6, 14, 15) and in his text.  

**Equivalency of stem-based and node-based trees**

   Below, we prove mathematically that node-based trees and stem-based trees carry the same information, albeit encoded in different ways.  We start by setting up some notation.

   Let *T* be a tree with root vertex *r*.  Recall that specifying a root for a tree determines its “parent” and “ancestor” relations completely.  If *x* is the parent of *y*, we will denote the edge joining them by the symbol *x*→*y* (in keeping with the convention that edges point from parents to children).  Alternately, we will write *x > y* to indicate that vertex * x* is an ancestor of vertex *y*.

   It is a standard fact that for every set *X *of vertices in *T*, there is a unique vertex *y* (which may or may not belong to *X*) with the following two properties: first, *y ≥ x* for every *x *in *X*, and second, if *z* is any other vertex such that *z ≥ x* for every *x *in *X*, then *z > y*.  The first of these conditions says that * y* is a common ancestor of the vertices in *X*; the second condition says that it is the *youngest* common ancestor.

   Finally, we call *T* a *planted tree* if its root *r* has only one child.  (“Planted” is a more restrictive condition than “rooted”; every planted tree is necessarily rooted, but not vice versa.)

   We now describe an equivalence between two different kinds of labeled trees.  Let * n* be any positive integer, and let *T* be a rooted tree with * n* vertices, labeled 1, 2, …, *n*.  (Any of these may be the root of *T*.)  Construct a tree *U* from *T* according to the following algorithm.  

      *Algorithm A*

1. Create a new root vertex, labeled 0, and create a new edge 0→*r*, where *r* = root(*T*).
2. Label each edge * v*→*w* of this tree with the number *w*.
3. Erase the labels of the vertices.

  

   An example of the construction of *U* from *T* is shown in Fig. 3.  (The vertex labels are shown in black, and the edge labels in red.)  Note that *U* has *n*+1 vertices, hence *n* edges, which are labeled 1, 2, …, *n*.  A consequence of the construction is that *U* is always a *planted* tree, because its root (from which the label 0 was erased) has exactly one child, namely, *r* = root(*T*).

**Figure:**
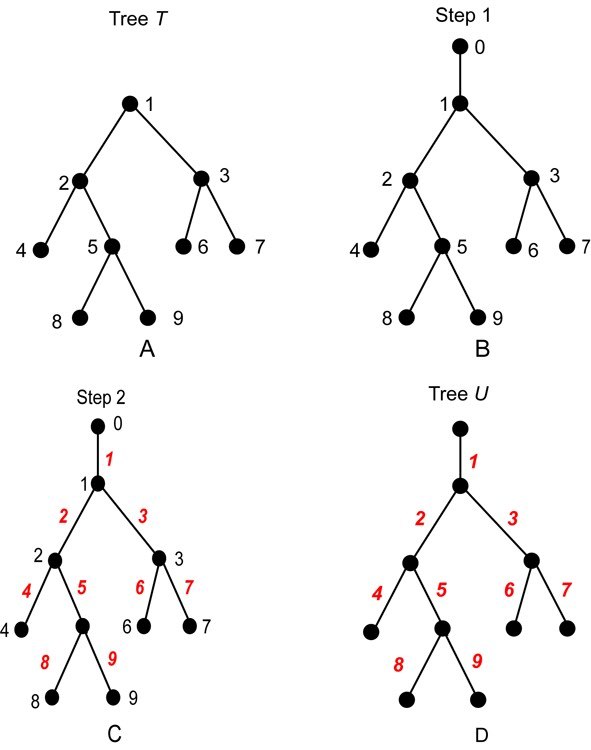
**Fig. 3. **The steps of Algorithm A, read A to D.  Reading D to A illustrates Algorithm B. 

We can reconstruct * T* from *U* by reversing Algorithm A.  Specifically, suppose that *U* is any planted tree with *n* edges, labeled 1, 2, …, *n*.  Note that *U* must have exactly *n*+1 vertices.  Let *r* be the root vertex, and let *s* be its unique child.  Now, construct a tree *T* from *U* as follows:  

      *Algorithm B*

1. Label each non-root vertex of *U* by the label of its parent edge, and assign the label 0 to vertex *r*.
2. Erase all labels on the edges.
3. Delete vertex * r* and edge *r*→*s*, and designate *s* as the root of the resulting tree.

  

   These steps are exactly the reverse of those of Algorithm A; for an illustration, see Fig. 3.  It is worth mentioning that the algorithms work the same way whether or not the input tree has polytomies (vertices with more than two children).  The algorithms establish the following mathematical fact.  

*Theorem 1*

   There is a one-to-one correspondence between the following two sets:

- The set of all rooted trees *T* on *n* vertices labeled 1, 2, …, *n*; and
- The set of all planted trees *U* on *n*+1 vertices, with edges labeled 1, 2, …, * n*.

   Because the correspondence is one-to-one, the rooted tree *T * contains exactly the same information as its planted counterpart * U*.  However, one must be careful when translating between * T* and *U*.  For example, there is *not* a one-to-one correspondence between arbitrary subtrees of *T* and arbitrary subtrees of *U*.  Indeed, if *E* is the set of edges of a subtree of *U*, then the corresponding set of vertices of * T* will not form a subtree unless *E* is planted.  For example, suppose that *T* and *U* are as shown in Fig. 3A and Fig. 3D, respectively.  The edges 4, 5, 8, 9 form a subtree of *U*, but vertices 4, 5, 8, 9 do not form a subtree of *T*; see Fig. 4A, B.  On the other hand, vertices 2, 4, 5, 8, 9 do form a subtree of *T* because the corresponding edges 2, 4, 5, 8 and 9 form a *planted* subtree of *U*; see Fig. 4C,D.

**Figure:**
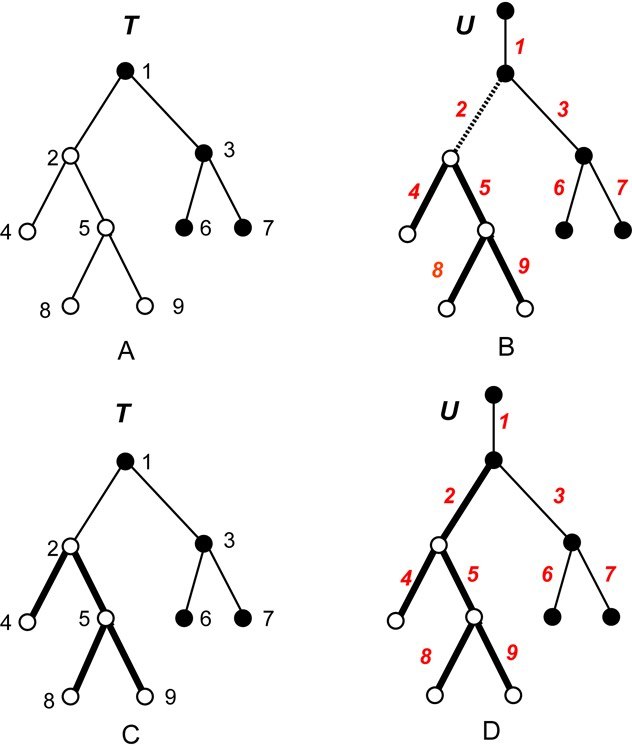
**Fig. 4.** In the node-based tree *T* (A), the vertices 4, 5, 8, 9 do not form a subtree, even though edges 4, 5, 8, 9 form a subtree of the corresponding stem-based tree *U* shown in B. In contrast, the subtree of *T* (C) formed by vertices 2, 4, 5, 8, 9, corresponds to the planted subtree of *U* shown in D. The figure also illustrates possible circumscriptions of the terminal taxa 4, 8, 9; heavy lines denote edges included in the classification. Applying node-based circumscription to the stem-based *U* results in the polyphyletic group of 4, 8, 9, and the inferred ancestor 5; as shown in B, there is no edge connection to the sister group comprising the terminals 6 and 7 because inferred ancestor 2 remains excluded (dashed line).  In contrast, a node-based circumscription of *T* or a stem-based circumscription of *U* (shown in C and D) yields the monophyletic group composed of the terminal taxa 4, 8, and 9 and their inferred ancestors 2 and 5.

Indeed, it follows from Algorithms A and B that there is a one-to-one correspondence between proper subtrees of *T* and planted proper subtrees of *U*.  Similarly, there is a one-to-one correspondence between subtrees of *T* (not necessarily proper) and planted subtrees of *U* (again, not necessarily proper).

   Additional biological information associated with a stem-based or node-based tree can be translated via this algorithm.  For instance, the character data represented by edge labels in a stem-based tree (Fig. 1B) can be represented by vertex labels in the corresponding node-based tree.   

**An example: node- and stem-based concepts of monophyly**

   While node-based and stem-based trees carry the same basic information about taxa and ancestry, they represent this information in different ways.  Therefore, it should not be surprising that biological concepts are modeled by different mathematical substructures in the two kinds of trees. We provide an example of this through a discussion of how the phylogenetic concept of monophyly is represented in each tree model. Hennig’s ([14], pp. 206–209) discussion of monophyly admits only one definition of this term; a monophyletic group is a group that includes all descendants of a common ancestral species. Although not mentioned in this section, elsewhere Hennig ([14]:71) makes it clear that he intends the ancestral species also to be a member of the group (and, indeed is logically equivalent to all descendant members of the group). Recently, additional means of circumscribing monophyletic groups were proposed ([18] [19] [20] [21]), which have now been codified into formal rules distinguishing several kinds of clade recognition. Two of these are germane to our discussion.

   Definition 1: “A node-based clade is a clade originating with a particular node on a phylogenetic tree, where the node represents a lineage at the instant of a splitting event.” (The PhyloCode version 4c, January, 2010, Article 2.2, [22])

   Definition 2. “A branch-based clade is a clade originating with a particular branch (internode) on a phylogenetic tree, where the branch represents a lineage between two splitting events.” ([22])

   We argue that this distinction between node-based and branch-based (= stem-based) concepts of monophyly arises from confusion between the two types of trees we have discussed.  This is not intended as a critique of the entirety of the PhyloCode, but rather is provided as an example of how being explicit regarding graphical models can provide clarity to discussions of biological concepts. Indeed, given the discussion of these tree models above and adopting Hennig's ([14], p.71) usage of “monophyly”, it is evident that a monophyletic group with common ancestor *A* is represented in a node-based tree *T* by the proper subtree *rooted* at the *vertex* corresponding to * A*, and in a stem-based tree *U* by the proper subtree *planted* at the *edge* corresponding to *A*.  Recall that the proper subtrees of *T* are in bijection with the planted proper subtrees of *U*.  To rephrase this observation, the correct mathematical representation of monophyly can be found either by applying Definition 1 to a node-based tree, or by applying Definition 2 to a stem-based tree. A node-based name cannot exist for a stem-based tree just as a stem-based name cannot exist for a node-based tree. If it is agreed that a tree must be either a node-based or a stem-based tree and not some mix of the two, then one must select the appropriate naming scheme to represent monophyly. While authors might argue for employing both concepts of monophyly for a single phylogeny, they must then recognize that such a phylogeny would not be a valid mathematical representation of a tree.

   It is worth examining what happens if we apply Definitions 1 and 2 to the wrong kinds of trees.  First, a “node-based clade” of a stem-based tree—speaking mathematically, a proper but non-planted subtree of a stem-based tree—does *not* correspond to a monophyletic group of taxa.  Returning to the phylogenetic tree *U* shown in Fig. 3D, the non-planted subtree highlighted in Fig. 4B is actually polyphyletic, not monophyletic; every edge in *U* represents a taxon descended from taxon 2, which does not belong to the subtree.  That this set of taxa is polyphyletic is perhaps clearer upon examining the corresponding vertices in the node-based tree (see Fig. 4A.)  This matches the definition of “crown clade”.  Second, a planted subtree in a node-based tree (such as the tree spanned by the black vertices 1, 3, 6, 7 in Fig. 4A) is not monophyletic but paraphyletic, because it includes only one child (3) of its root vertex while excluding child 2 and the children of 2). It is tempting to interpret such a tree as a stem-based clade that includes a “root edge”—here the edge from 1 to 3—but not its parent vertex, here 1.  However, the mathematical definition of a graph does not permit such a structure; one cannot have an edge without both its endpoints. Omitting the “root edge” produces a well-defined graph that contains the same biological information (regarded as a node-based tree). If we are careful only to use the term “node-based clade” when working with node-based trees, and “stem-based clade” when working with stem-based trees, then the two terms become synonymous. The difference has no biological significance and lies only in the form of tree chosen to represent the phylogeny. Both node-based and stem-based names as proposed in the PhyloCode describe the single concept of monophyly, albeit based on two possible tree graphs. Given that in empirical phylogenetic studies all recognized monophyletic groups must be corroborated by one or more synapomorphies (though not necessarily unique and unreversed), we suggest that the PhyloCode be amended to reflect this. A simple approach would be to state explicitly in the PhyloCode one of the two graphical representations of trees for reference and then apply the logical corresponding concept of monophyly throughout the PhyloCode.  

**Conclusion**

   Practicing "tree-thinkers" might easily make the mental conversion between node-based and stem-based trees. By explicitly detailing that these tree models are mathematically equivalent, we aim to add clarity to discussions related to the biological meaning of phylogenies. It is important to be specific about these two distinct representations of trees. During the latter half of the twentieth century, phylogenies transitioned from being essentially cartoon-representations to graphical representations of the results of an analysis of data (typically represented in a matrix). We argue that biological concepts relating to a phylogeny that is inferred based on an analysis of data should be discussed in a context consistent with the graphical model used to display results of the analysis. To our knowledge, most evolutionary biologists do not construct estimates of phylogenetic relationships based on mathematical models in which transformations of characters occur at *both* nodes and along branches. Instead, computations are made at either vertices (= nodes) or edges (= stems). We leave open the possibility that authors might employ a workable mental model in which character transformations occur along both nodes and branches, but we argue that this would not be strictly representing the results of the analysis. Last, and importantly, we add that representing relationships between taxa via either a node-based or stem-based tree does not preclude subsequent use of the same phylogeny to model processes that might occur along both nodes and branches (as implemented, for example, in the dispersal-extinction-cladogensis model of geographic range evolution; [23] [24]). Without clear recognition of node-based and stem-based trees, as well as the equivalency between these, authors may arrive at confused interpretations of phylogenies, including circumscriptions of monophyly.   

**Acknowledgements**

    EOW thanks the late David Hull (Northwestern University) for sending a copy of a manuscript that he never published entitled “Hierarchies and Hierarchies” that touched upon the problems associated with process/pattern and tree/cladogram controversies, and for what must have seemed to him hours of discussion on things phylogenetic and philosophical regarding the subject. We also thank Shannon DeVaney (Los Angeles County Museum) and Mark Holder (University of Kansas) for reading the manuscript and providing a critical review.   

**Funding Information**

JLM was partially supported by NSA Young Investigators Grant #H9B230-08-1-0073. DCB Partial support to EOW by NSF DEB 073289, the Euteleost Tree of Life project, which includes a component aimed at increasing understanding of phylogenetic trees, is gratefully acknowledged. 

**Competing Interests**

The authors have declared that no competing interests exist.
